# Building bio-innovation systems through advanced biotechnology education

**DOI:** 10.3389/fbioe.2024.1415103

**Published:** 2024-07-09

**Authors:** Edgar Cardozo Ruíz Díaz, Silverio Andrés Quintana, Cinthia Mabel Rojas, Danilo Fernández Ríos

**Affiliations:** ^1^ Departamento de Biotecnología, Facultad de Ciencias Exactas y Naturales, Universidad Nacional de Asunción, San Lorenzo, Paraguay; ^2^ Mycology Investigation and Safety Team, Centro Multidisciplinario de Investigaciones Tecnológicas, Universidad Nacional de Asunción, San Lorenzo, Paraguay

**Keywords:** sustainable development, stakeholder integration, virtual learning, biotechnology policy and regulation, innovation and entrepreneurship

## Abstract

We discuss the role of advanced biotechnology education in fostering sustainable bio-innovation systems. As a case study, we focus on Paraguay’s Graduate Diploma in Innovation Management and Biotechnological Projects, which emphasizes interdisciplinary collaboration, stakeholder integration, and professionals skilled in the interplay between biotechnology, society, and governance. We highlight the relevance of educational programs in addressing the gap between academic research and industrial needs, thereby contributing to sustainable growth in the biotechnology sector.

## 1 Introduction

To address global challenges such as climate change and food security, a sustainable bio-innovation system is essential. This involves developing solutions that promote economic growth while minimizing environmental impacts, addressing social concerns, and engaging in participatory governance (McCormick and Kautto, 2013; Falcone et al., 2019; Eckardt et al., 2023).

In the last 10 years, with the support of the National Council of Science and Technology (CONACYT), Paraguay has started to develop advanced educational programs with the objective of generating professionals ready to tackle these challenges (Delgado, 2023). Significant resources have been used to develop biotechnology-related programs, and one of their strategic axes is to support projects related to bio-innovation.

An efficient education system provides the necessary knowledge and skills to make informed decisions regarding the development and application of new technologies (Steele and Aubusson, 2004). This is essential for comprehending the intricacies of biotechnology, including business aspects, intellectual property protection, and regulatory environments (Narasimharao, 2010). Furthermore, thorough education fosters biotechnological literacy, which is becoming increasingly essential in contemporary society, due to significant advancements in agriculture, industry, and medicine (Mohd Saruan et al., 2015; De La Hoz et al., 2022).

## 2 How can a curriculum be developed for a bio-innovation course?

### 2.1 Aspects that should be considered

**FIGURE 1:**
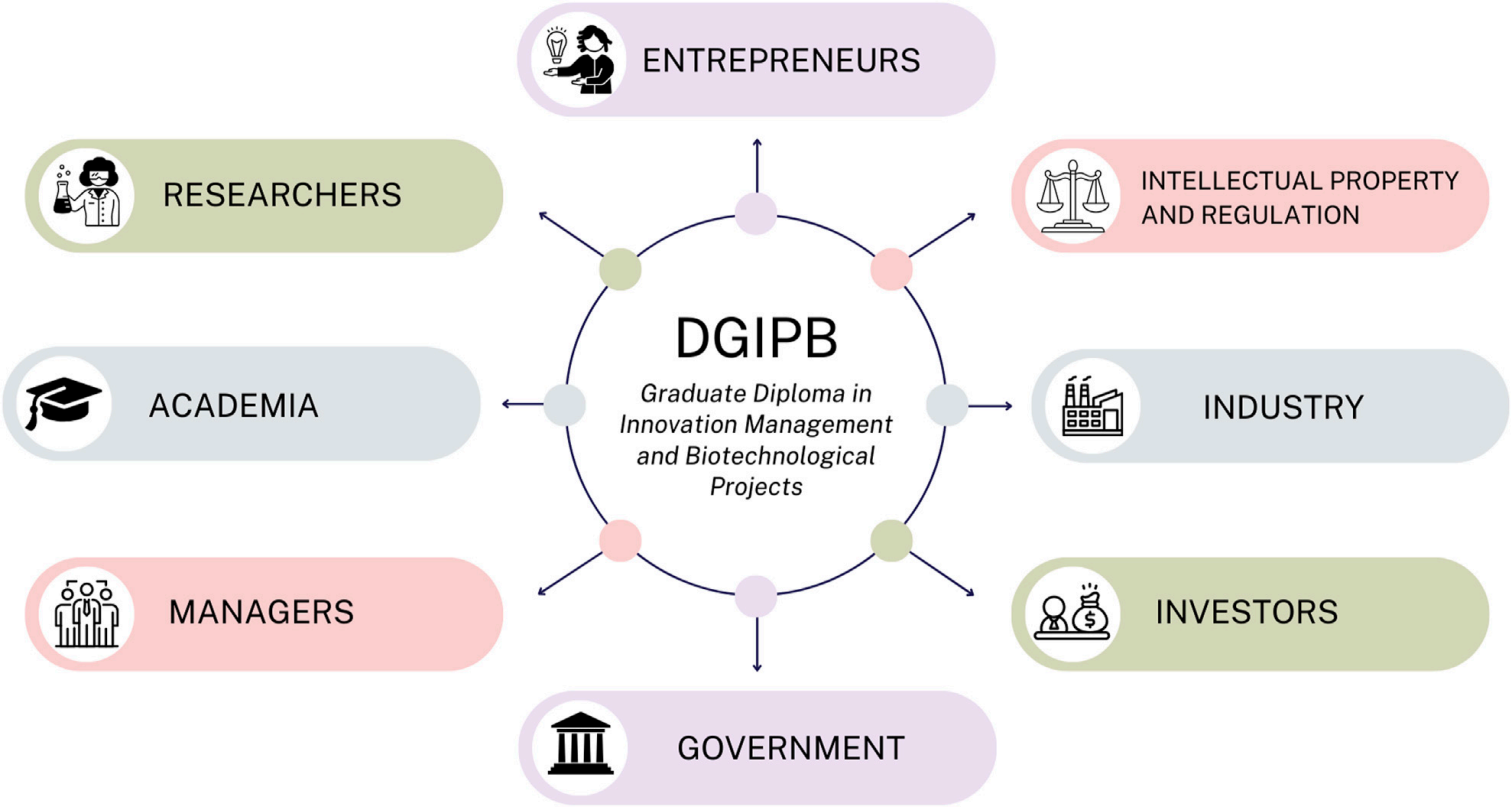
Stakeholder Integration in the Graduate Diploma in Innovation Management and Biotechnological Projects (DGIPB). This figure illustrates the DGIPB stakeholder network, reflecting the multidisciplinary collaboration that is essential for advancing bio-innovation.

The development of an educational program focused on bioinnovation requires planning that considers several factors. For this purpose, we will take the case of a Graduate Diploma in Innovation Management and Biotechnological Projects (DGIPB) developed by the Faculty of Exact and Natural Sciences (FACEN) at the National University of Asunción (UNA) as an example to address the need for biotechnological applications that can solve identified problems and capitalize on opportunities (CONACYT, 2023). This program aligns research outcomes with practical issues, facilitating the development and adoption of bio-innovative strategies while also considering economic factors. The primary goal of the DGIPB is to build bridges between academia, business, regulation, and civil society, ultimately facilitating the development and adaptation of technologies for their transfer to the productive and social sectors through the assistance of *Innovation Managers* (Figure 1).

The Diploma, which was co-funded by FACEN and CONACYT through the Innovation Program in Paraguayan Companies (PROINNOVA), explicitly focuses on biotechnology. The program is designed to equip professionals with the ability to combine scientific, economic, and legislative understanding to recognize the context of biotechnological development initiatives. Furthermore, it offers guidance in identifying the intricacies of international and domestic legislation concerning intellectual property protection.

The DGIPB curriculum includes three modules.

1. Fundamentals of bioeconomy and innovation: This module examines the dimensions and aspects that define biotechnology development systems, providing a comprehensive overview of the industry, including its technology, growth prospects, and economic development. In addition, this module addresses the key elements that impact activities related to the bioeconomy and innovation ecosystems, including public policies and regulatory frameworks.
2. Innovation management and biobusiness: This module is designed to help participants understand the nature of managing innovation in the biotechnology sector and develop appropriate business models that can leverage the benefits of an innovation management system.
3. Formulation of biotechnology projects: The final module focuses on identifying key variables and actions involved in the conception, planning, execution, and evaluation of biotechnology projects. This module culminates in the formulation of an innovative biotechnology project that serves as the capstone of the course.

While there are similar programs in the country that focus on project management and innovation, two factors make the DGIPB unique. First, none of them specializes in bioprojects. This highlights its relevance in the training of professionals in the Paraguayan context. Second, as the regulatory environment has a significant impact on the direction and speed of innovation, the DGIPB also focuses on public policies and regulatory frameworks that govern various biotechnology industries. Unfortunately, this aspect is frequently disregarded in similar courses, which can have severe repercussions on the innovation landscape (Bogner and Torgersen, 2018).

One of the features of the DGIPB is that it was conducted entirely virtually, which facilitated interaction between students and professionals who were geographically distant from one another. Virtual education has been demonstrated to be effective as a flexible and time-efficient means for students to receive guidance (McReynolds et al., 2020), enabling them to establish multiple relationships and seek advice from the entire network during the program. Furthermore, to promote and encourage enrollment, CONACYT provided economic support to organize the DGIPB and full scholarships for all admitted students, granting them the opportunity to attend at no cost.

### 2.2 Is it possible to measure the course’s impact on bio-innovation?

Nineteen students completed the DGIPB in the first edition of the program. Among them, nine belonged to academic institutions, such as universities and research centers; six worked in the private sector for companies involved in pharmaceuticals and agro-inputs; and two were members of the government sector. Students were given the opportunity to create, develop, and refine Research and Developments and Innovation projects (from now on R&D + i) that focused on the application of biotechnology and introduction of bioproducts to the market. Through this process, they were able to identify the key factors involved in each stage of a biotechnology project, and use analytical tools to assist in planning, controlling, and managing these projects.

Nine projects were developed within the DGIPB framework, three of which were presented at the III Paraguayan Biotechnology and Applications Conference. This event, directly linked to the DGIPB as a graduation act, was themed “Innovation and Bio-business,” and featured participants from the Ministry of Industry and Commerce, Paraguayan Industrial Union, and private investors focused on Science, Technology and Business (STBs). The initiative was supported by Technological Linkage Managers from the National University of Rosario and the National University of San Martin in Argentina as well as company executives and investor groups from GeneBiome EAS, Paraguayan Association of Venture Capital (PARCAPY), Paraguayan Innovation Investment Fund (FIIP), OpenX in Paraguay, GRIDX in Argentina, and Bioeutectics in Argentina and the United States. The expertise of these individuals and organizations is crucial for the development of a scientific business ecosystem in Paraguay (FACEN, 2023).

The primary focus of projects initiated by DGIPB graduates was on the development and delivery of biotechnology-based goods and services (OECD and Eurostat, 2018a). These projects emphasized R&D + i and entrepreneurial endeavors. One such project, led by a graduate working in the government sector at the Ministry of Industry and Commerce, focused on innovation management. This area involves all-encompassing activities essential for planning, governing, and managing resources to foster innovation (OECD and Eurostat, 2018b). This project also diverged from the typical trajectory pursued by natural science professionals, who generally focus on the technical aspects of product development. This divergence highlights a distinct application of the skills and knowledge acquired through the DGIPB programme, thereby underscoring its versatility.

After completing each module, surveys were conducted among the students to obtain feedback, which was subsequently used to make improvements (Table 1). These results demonstrated that the DGIPB was highly appreciated. Students expressed satisfaction with the content and valued their interactions with actors in the regional innovation environment. The DGIPB facilitated a stronger connection between the academic and business sectors, highlighting potential commercial applications rooted in scientific and technological advancements.

**TABLE 1:** Outcomes and project engagement of the DGIPB graduates.

Indicator	Degree of compliance (%)	Lessons learned
Percentage of graduates whose subsequent professional practice is linked to innovation management and/or biotechnological projects	70	Graduates remain connected to innovation and/or biotechnological projects, suggesting a link between their professional endeavors and the DGIPB. This continuity highlights the relevance and practical applications of the course
Tools or skills acquired in the DGIPB for professional practice	100	There is a primary interest in planning and management tools as well as in financial administration. The experiences of expert guests (entrepreneurs and investors) were valued
Percentage of graduates who participated in at least one project after completing the DGIPB	62	The graduates participated in at least one project after completing the DGIPB
Percentage of new projects generated by graduates after completing the DGIPB	54	Almost half of the graduates generated at least one new project after completing the DGIPB
Percentage of projects associated with public entities	77	Most projects are associated with the public sector and are funded by research funds. More emphasis should be placed on opportunities for private investment
Percentage of projects linked to foreign companies or institutions	15	Only two projects showed links to foreign capital. Emphasis should be placed on private foreign capital investment opportunities

The interactions that took place between professors, mentors, guest speakers, and students were very rich and fruitful in the DGIPB. Professionals from outside academia were eager to collaborate with the program by sharing their experiences through discussions, reflecting the urgency of developing a collaborative system for bio-innovation.

## 3 Biotechnology ecosystem for sustainable bio-innovation

Paraguay has been identified as one of the countries with the highest growth projections (IMF, 2021). Despite the COVID-19 pandemic, macroeconomic stability remains appealing to investors. However, the percentage of Gross Domestic Product (GDP) allocated to R&D + i in Paraguay is significantly lower than the regional average (0.15% in 2022 compared to 0.61% in Latin America and the Caribbean). Nevertheless, the public sector is the primary source of funding, and primary efforts to conduct R&D + i activities are led by universities and public agencies (CONACYT, 2024).

Paraguay’s latest socio-economic development strategy, outlined in the IICA Bioeconomy proposal, calls for the expansion of biomass supply and the creation of sustainable conditions for its reproduction. Additionally, the plan aims to add value to local production, give businesses a competitive edge in the global bioindustry market, and establish connections with dynamic global markets (Productiva, 2022).

Although Paraguay has established a regulatory framework for biotechnology that can facilitate locally developed products (Benitez Candia et al., 2024), the country’s scientific–technological ecosystem, consisting of public and private research institutions, universities, and funding organizations, does not seem to provide complete support for local development. While all technological projects are evaluated in terms of R&D + i costs, regulatory aspects, potential benefits, prospective markets, and possible degree of adoption, sufficient funding is often unavailable to advance and complete final product development. This phenomenon also occurs with products obtained through new breeding technologies (NBTs), which may be more readily implemented because of their streamlined regulatory procedures (Fernández Ríos et al., 2024).

Considering this scenario, FACEN has established inter-institutional cooperation agreements with various organizations to enhance education, research, and technological development. These agreements facilitate collaboration between domestic and international companies and institutions, promoting strategic alliances to train highly qualified entrepreneurs. This is crucial for addressing the challenges outlined in Paraguay’s Vision 2030, which aims to transform the economic model into a knowledge-based one (STP, 2014).

Throughout the DGIPB, investors identified the disconnection between academia and the production sector as a significant issue. The lack of coordination, information, communication, and articulation among different innovation actors is deemed a critical problem. For instance, the DGIPB facilitated discussions between a company led by a diploma graduate and FACEN, with the aim of developing local bioproducts. However, it was observed that many companies in the production sector are still unaware of the existence of biotechnology professionals in the country, resulting in them seeking foreign advice.

This type of collaboration between public and private sectors has historically been viewed as a strategy for enhancing research responsiveness to evolving global challenges, thereby expediting innovation and facilitating broader economic and social benefits from joint investments made by governments and private industries. Furthermore, these collaborations can facilitate the alignment of academics’ specialized knowledge with the skills of industry scientists, thereby translating scientific advancements into practical applications within a stable funding environment (Gloger et al., 2021).

## 4 Discussion

Considering a regional scenario, prior to 2001, scientific research and development in Latin America had not been a priority for governments, in the same manner as in similar-sized economies. However, a period of sustained investment followed with budget allocations for state research agencies and the formation of new Ministries for Science. Additionally, funding was provided for international training programs, leading to an increase in scientific output and the return of scientists who had previously been based overseas to lead research initiatives in their home countries. This investment demonstrates the potential benefits of shifting towards a knowledge-based economy (Catanzaro et al., 2014; Van Noorden, 2014).

Experiences such as GlaxoSmithKline (GSK) introduced a pioneering model for public-private collaborations called the *Trust in Science* initiative (GSK, 2018). Initially concentrated in Argentina and Brazil, this initiative was later expanded to include Uruguay and Mexico. The model stands out because of its emphasis on direct scientific collaboration between academic and industrial scientists, its specialized approach to intellectual property rights for academic collaborators, and its transparent process for project applications and joint funding decisions with governments (Gloger et al., 2021).

*Trust in Science* has also taken steps to ensure that its advantages extend beyond financial support to encompass mentorship, project guidance and direction, and fostering local scientific talent. This collaborative effort has provided professionals valuable experience in learning how their research ideas can be transformed into commercially viable products. In contrast, Paraguay has not yet reached the same level of success as its Mercosur counterparts and has not been involved in similar initiatives.

Despite the advantages of industry-academia partnerships, there are also challenges that must be considered. For instance, a sector of the academia views with apprehension the private sector´s sponsored research as market driven, at the cost of basic science and academic freedom (Palmer and Chaguturu, 2017). Furthermore, concerns regarding intellectual property rights may arise, emphasizing the importance of addressing these issues to ensure long-term sustainability. Bio-innovation has the potential to affect socioeconomic development significantly, underscoring the need for a skilled workforce to guide progress. Educational programs that provide a solid foundation in biotechnology management can potentially bridge the gap between academia and industry, ensuring that graduates are adequately prepared to fulfill the requirements of the biotechnology sector. This alignment between education and industry demand is critical for the sustained growth and success of the biotechnology industry.

## Data Availability

The original contributions presented in the study are included in the article, further inquiries can be directed to the corresponding author.
